# MALIGNANT TRANSFORMATION OF ACTINIC KERATOSES TO SQUAMOUS CELL CARCINOMA IN AN ALBINO

**DOI:** 10.4103/0019-5154.48986

**Published:** 2009

**Authors:** Vijaya Sivalingam Ramalingam, Ramapriya Sinnakirouchenan, Devinder Mohan Thappa

**Affiliations:** *From the Department of Dermatology and STD, Jawaharlal Institute of Postgraduate Medical Education and Research (JIPMER), Pondicherry, India*

**Keywords:** *Albinism*, *actinic keratoses*, *cutaneous horns*, *squamous cell carcinomas*

## Abstract

A 25-year-old male, who was a known case of oculocutaneous albinism presented to us with right inguinal swellings of six months' duration. He gave a preceding history of a similar lump in the right thigh, which was excised at the Chennai Government Hospital. He was diagnosed to have oculocutaneous albinism with actinic keratoses, with multiple squamous cell carcinomas (with metastatic deposits in the right inguinal region) and cutaneous horns. The case is reported to highlight preventive aspects in the management of albinos.

## Introduction

Albinism is inherited as an autosomal recessive disorder, characterized by lack of skin pigment, as a result of which albinos are highly susceptible to sun-induced damage to skin.[1] Its estimated frequency is 1: 20,000 in most populations. Its incidence is higher among the populations close to the equator, one of the highest being among the Cuna Indians, 63 per 10,000 population. Previous studies have documented a high frequency of actinic keratoses and skin cancers in albinism patients.

Albinism is broadly divided into two types: ocular albinism and oculocutaneous albinism.[2,3] It is a disorder characterized by nystagmus, strabismus, photophobia, foveal hypoplasia and decreased visual acuity. These are the ocular problems faced by albinos. The cutaneous problems seen with oculocutaneous albinism are sun burns, basal cell carcinoma, malignant melanoma, dysplastic nevus syndrome and, most important and most common of all, actinic keratoses predisposing to squamous cell carcinoma.[4]

A correlation has been established between exposure to sun and the incidence of the above cutaneous problems in oculocutaneous albinism.[1] Albinos are at an increased risk of developing skin malignancies and, hence, a regular examination for early detection and treatment of the malignancies would increase their life expectancy to a great extent. We report here a case of oculocutaneous albinism with multiple actinic keratoses transforming into squamous cell carcinoma and cutaneous horns.

## Case Report

A 25-year-old male, who was a known case of oculocutaneous albinism, presented with right inguinal swellings of six months' duration. He gave a preceding history of a similar lump in the right thigh, which was excised at the Chennai Government Hospital. On further interrogation, he gave a history of asymptomatic keratotic growths over the trunk and limbs, of one and a half years' duration. He also gave a history of photosensitivity and presence of white hair since birth.

**Figure 1:**
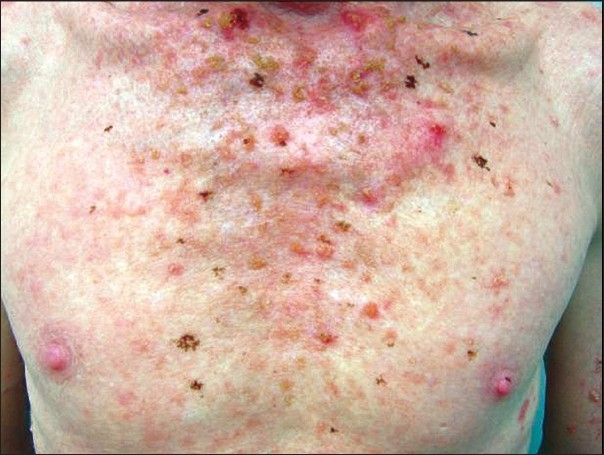
Albino showing actinic keratoses lesions over the chest

On physical examination, he had typical features of oculocutaneous albinism in the form of totally depigmented skin with white hairs. He had photophobia, marked nystagmus and decreased visual acuity. His iris was non-pigmented and the pupillary area looked red. He had multiple keratotic papular and plaque lesions of 2mm to 2cm in size [Figure 1], that were round to oval in shape, surrounded by a zone of erythema (actinic keratoses). Besides this, he had ulcerated and crusted lesions, up to 12 in number.

**Figure 2:**
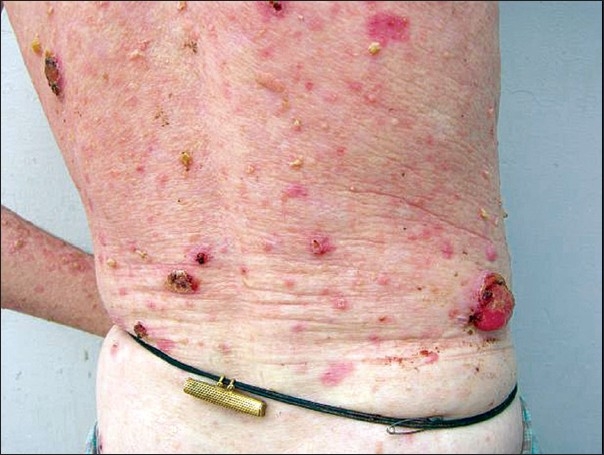
Malignant transformation of actinic keratoses to squamous cell carcinoma

**Figure 3:**
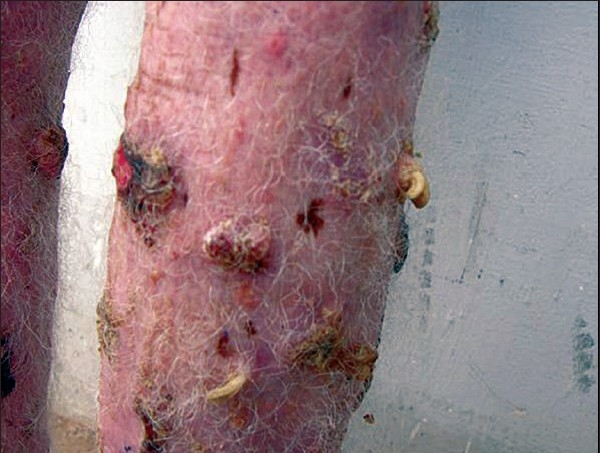
Lower leg developing cutaneous horns

These ulcerated areas were indurated and some of them showed exophytic growths [Figure 2]. In the right leg, horny excrescences [Figure 3] were seen over the keratotic lesions (cutaneous horns). The height of these horns was around 1.5cm.

On the basis of these clinical findings, a diagnosis of oculocutaneous albinism with actinic keratoses with multiple squamous cell carcinomas (with metastatic deposits in right inguinal region) and cutaneous horns was made.

The hemogram showed hemoglobin of 9 gm%. His total leukocyte count, differential leukocyte count, and platelet counts were within normal limits. Fine needle aspiration cytology (FNAC) from the right inguinal region showed features of poorly differentiated squamous cell carcinoma. During follow up, it was found that he had been receiving radiotherapy (RT) in the right inguinal region for the past four months, with partial response. The overlying skin had healed, but still an indurated mass of about 2cm could be made out. He was also given chemotherapy with cisplatin and 5-FU. Recently, he had been admitted to the surgery ward for excision of malignancies.

## Discussion

Oculocutaneous albinism (OCA) is the most common inherited disorder of general hypopigmentation, with an estimated frequency of 1: 20,000, in most populations.[1] There are several types of albinism.[2] Tyrosinase-related OCA (OCA1), one of the two most common types of albinism, is produced by loss of function of the melanocytic enzyme tyrosinase, resulting from mutations of the tyrosinase gene. Null mutations result in total loss of function and no pigment formation (OCA1A), whereas ‘leaky’ mutations result in an enzyme that retains some function and, therefore, some pigment formations is seen (OCA1B). A typical feature of OCA1 is ‘white hair’ at birth.[2,3] The case in study belonged to this category. From the clinical features, it was obvious that the patient fell under the subtype OCA1A or classic tyrosinase-negative OCA resulting in the characteristic ‘albino’ phenotype.

In OCA patients, lack of melanin, a photo protective pigment against the harmful UV radiation, predisposes to actinic keratoses and, thereby, to squamous cell carcinomas.[1] It is estimated that the risk of skin cancer is inversely related to the distance from the equator. This is depicted by the early age of onset of skin cancer among the albinos, who live close to equator. Males develop actinic keratoses more frequently than females.[5] They usually occur on sun exposed areas. Actinic keratoses is a clinical manifestation of UV radiation (most commonly UV-B radiation) induced neoplastic transformation of keratinocytes.[5,6] The UV-B radiation causes thymidine dimer formation in DNA and RNA, resulting in mutations causing neoplastic changes in keratinocytes. The two important sites of mutations taking place in actinic keratoses formation are in telomerase and the tumor suppressor gene p53, located on chromosome 17p132. These mutant DNA cells, resistant to apoptotic death, undergo clonal expansion and accumulate genetic injury, resulting eventually in neoplastic transformation.[5,7]

Studies have shown that up to 60% of the squamous cell carcinomas begin as actinic keratoses and that there is histologic evidence of contiguous actinic keratoses in 97% of the squamous cell carcinoma lesions that arise on sun damaged skin.[5] The likelihood of a fully developed squamous cell carcinoma evolving from a given actinic keratosis has been estimated to occur at a rate of 0.075-0.096% per lesion per year. Thus, an affected individual, with an average number of 7.7 actinic keratoses on his skin, could expect to develop squamous cell carcinoma at a rate of 10.2% over 10 years. Other sources give even higher estimates, with rates of 13-20% of such individuals developing squamous cell carcinoma over a 10-year period and with the albinos being even more vulnerable, due to lack of photo protective pigment.[5]

A variety of treatment options exist for actinic keratoses.[5] The most common ones are cryotherapy with liquid nitrogen, 5-fluorouracil and curettage. The interferon inducer imiquimod can also be used. Topical chemotherapeutic agents, such as those containing 5-fluorouracil, are used commonly in patients with multiple lesions. Less commonly, dermabrasion, excision, chemical peel, laser therapy, or photodynamic therapy is used.[5]

Treatment of actinic keratoses and skin cancers is important, but skin cancer prevention is an even more important long term goal.[8] This can be accomplished only by avoiding the sun and/or by using sun protection methods. Avoiding the sun is difficult to achieve among the rural communities, since most of them work outdoors. But it is recommended that it would be best if the albinos were employed indoors. Sun protection measures need to be directed to the areas of the skin most susceptible to actinic damage like the head, neck, lips, hands, arms, legs etc. Some of the measures include using large broad brimmed hats, long sleeved shirts and long trousers or skirts as uniforms for albino students, regular use of sunscreen lotions on exposed skin and, above all, educating the albinos about the harmful effects of exposure to sun.[8]

Squamous cell carcinoma, of which the solar keratotic type is by far the most common, is the most populous of all cutaneous malignant neoplasms.[9] No one can predict which specific actinic lesion will progress to squamous cell carcinoma or which particular patient will develop metastatic squamous cell carcinoma. Since squamous cell carcinoma is a frequent cutaneous neoplasm and it causes significant morbidity and mortality, actinic keratoses should be treated with the same respect given to other epithelial neoplasms, such as those of the uterine cervix and oral mucosa.[5]

Currently an estimated 1200 persons die of metastatic squamous cell carcinoma each year and now the freedom that physicians have to eradicate these lesions early in their evolution affords the opportunity to reduce that figure significantly.[5] Albinos need protection against their major enemies - the neglect of an uninformed society and the hostility of the tropical sun. Registering all albinos early in life, educating the society on the educability of most albinos with correctable ocular defects and offering them encouragement and guidance through school and employment will help to restore them in society. The damaging effect of the sun can be minimized by using protective clothing, sun screening agents and indoor occupation.[5]

Regular examination of all albinos for early detection and treatment of the various malignant lesions to which they are prone deserves to be included in the current anti-cancer campaign, to which the medical world is committed.[2,3] Genetic counseling could also be adopted. Physicians should maintain a high index of suspicion in this vulnerable population.
